# Dielectric Properties of Dual-Frequency Reactive Mesogens before and after Photopolymerization

**DOI:** 10.3390/ma7021113

**Published:** 2014-02-11

**Authors:** Takayuki Kumagai, Hiroyuki Yoshida, Masanori Ozaki

**Affiliations:** 1 Division of Electrical, Electronic and Information Engineering, Osaka University, 2-1 Yamada-oka, Suita, Osaka 565-0871, Japan; E-Mails: tkumagai@opal.eei.eng.osaka-u.ac.jp (T.K.); ozaki@eei.eng.osaka-u.ac.jp (M.O); 2 PRESTO (Precursory Research for Embryonic Science and Technology), Japan Science and Technology Agency (JST), 4-1-8 Honcho Kawaguchi, Saitama 332-0012, Japan

**Keywords:** reactive mesogen, dielectric property, dual frequency, liquid crystal, photopolymerization, dielectric anisotropy

## Abstract

The dielectric properties of reactive mesogens before and after photopolymerization were investigated. Commercially available nematic reactive mesogens (RMS03-013C, Merck) were measured and found to be dual-frequency liquid crystals. The property arose from the δ-relaxation process that was caused by rotational fluctuations parallel to the molecule’s long axis. After polymerization, the polymerized reactive mesogens still exhibited this dual-frequency property. The result was attributed to the β-relaxation process which arose from rotational fluctuations of localized parts of the main chain. The sign change of the dielectric anisotropy with increasing frequency after polymerization was opposite to the sign change before polymerization.

## 1. Introduction

Reactive mesogens (RMs) are polymerizable liquid crystal (LC) monomers with rigid cores and reactive end groups. RMs can be easily aligned using the same methods used for low molecular mass LC. The end groups are polymerized with other end groups in the presence of photoinitiators and UV light, and form a film. Consequently, the orientation of the RM molecules is “frozen” and the film still has birefringence [1,2]. This remarkable characteristic makes them attractive as promising materials for light actuators such as polarizers, color filters, retarders and so on [3–5]. On the other hand, polymerized RMs (PRMs), which are stimuli-responsive materials, can be actuated by light [6–9], heat [10–12] and their properties have been well studied. There are also few reports about electric field driven PRMs [13,14]. However, investigation of the dielectric properties of PRMs in these studies were not carried out adequately and the dielectric constants of RMs before polymerization were used to investigate the kinetics, although the dielectric properties of PRM, in which the fluctuations of molecules is restricted, is supposed to be different from unpolymerized RMs [15–17]. Therefore, investigation of relations between the dielectric property and the behavior of PRMs as actuators is necessary to achieve more development in PRM actuator research.

In this study, we investigate the dielectric properties of commercially available nematic RMs (RMS03-013C, Merck, Darmstadt, Germany) in both the monomer and polymer state. The RMs were found to be dual-frequency LCs (DFLCs) in the monomer state. DFLCs change the sign of the dielectric anisotropy and are studied mainly for improving response speed of LCs [18]. It is of interest to examine how the dielectric properties of dual-frequency RMs change after polymerization.

## 2. Experiment

**Figure 1.**
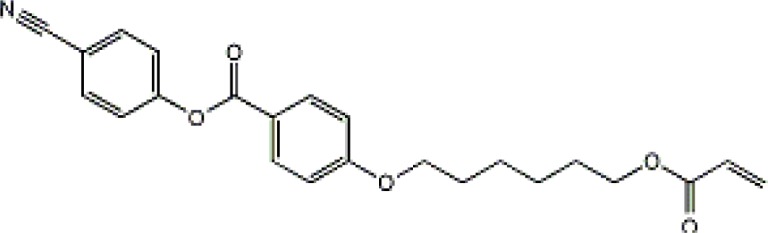
Chemical structure of 4-[6-(Acryloyloxy)hexyloxy]benzoic acid=4-cyanophenyl ester included in the sample.

The sample was prepared by mixing a photoinitiator [2-Benzyl-2-(dimethylamino)-4’-morpholinobutyrophenone, Tokyo chemical industry] with RMs at a concentration of 2.8 wt%. It should be note that the RM mixture contains several RMs and one of the RMs, 4-[6-(Acryloyloxy)hexyloxy]benzoic acid=4-cyanophenyl ester (see Figure 1), is pronounced. The glass substrates with indium tin oxide electrodes and alignment layers were used for assembling sandwich cells. Planar and homeotropic alignment were adopted to measure the dielectric constant perpendicular (ε_⊥_) and parallel (ε_||_) to the molecular long axis, respectively. Planar alignment polyimide (AL1254, JSR, Tokyo, Japan) and homeotropic alignment polyimide (JALS-20210-R2, JSR) were used; AL1254 was rubbed to induce uniaxial alignment. These cells were filled with the samples and PRMs were obtained by UV irradiation (25 mW/cm^2^, 60 s) at room temperature using a UV lamp (Lightningcure LC9566, Hamamatsu Photonics, Hamamatsu, Japan).

The impedance of the cells was measured at elevated temperature using an impedance analyzer (4294A, Agilent Technologies, Santa Clara, CA, USA). The amplitude of the probe signal is 0.02 V and the frequency range was varied from 10^3^ to 10^5^ Hz. The complex dielectric constant, ε = ε′ − *i*ε″, was calculated by representing the cells with an equivalent circuit consisting of a parallel connected resistance and capacitance. The dielectric anisotropy was obtained by the relation Δε = ε_||_ − ε_⊥_.

## 3. Results and Discussion

**Figure 2.**
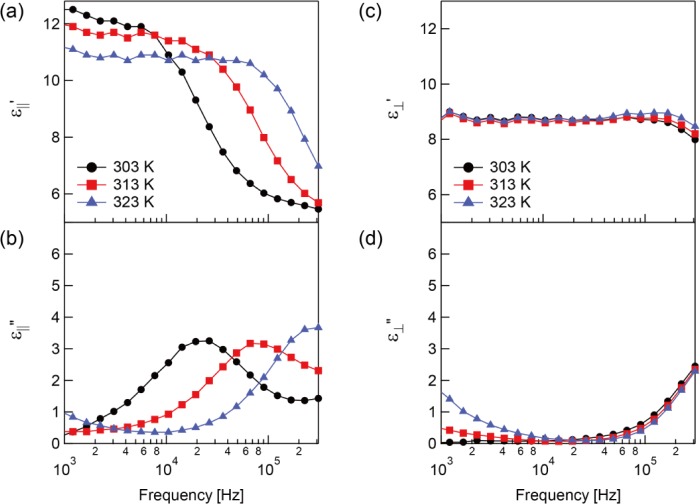
Measured complex dielectric constants before polymerization. The (**a**) real part; (**b**) imaginary part of complex dielectric constant parallel to the molecular long axis and the (**c**) real part and (**d**) imaginary part of complex dielectric constant perpendicular to the molecular long axis.

The dielectric constants before polymerization up to 323 K are shown in Figure 2. It should be noted that the phase transition of the sample from the nematic to isotropic state was at approximately 329 K. The relaxation process was observed and indicated by a steep decrease of ε_||_′ (Figure 2a) and a peak in ε_||_″ (Figure 2b). On the other hand, ${\varepsilon^{\prime }}_{⊥}$ and ${\varepsilon^{\prime \prime }}_{⊥}$ do not have a frequency dependence (Figure 2c,d).

Detailed information about relaxations can be obtained using the Cole-Cole plot by calculating the fitting model function of Havriliak–Negami [19] described below:

$$\varepsilon =\varepsilon_{\infty }+(\varepsilon_{s}-\varepsilon_{\infty })\frac{1}{{\left[1+{\left(i\omega \tau \right)}^{b}\right]}^{a}}$$ (1)

**Figure 3.**
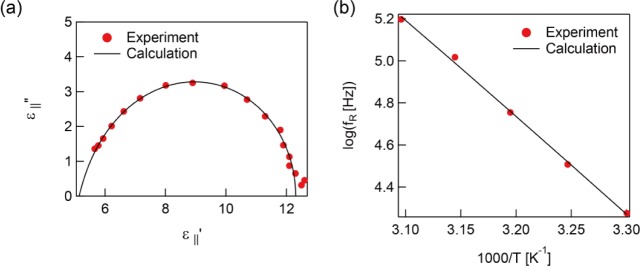
(**a**) The Cole-Cole plot of the unpolymerized sample at 303 K; (**b**) Temperature dependence of the relaxation frequencies of ε_‖_″ before polymerization. The filled circles are experimental data and the line is the calculated result.

Here, ω *=* 2π*f* is the circular frequency and τ is the relaxation time multiplied by 2π. The relaxation frequency *f*_R_ can be obtained by *f*_R_ = τ/2π. ε_s_ − ε_∞_ is the relaxation strength where ε_s_ and ε_∞_ are the dielectric constant at the extra low and high frequency, respectively. *b* and *a* are shape parameters (0 < *b*, *ab <* 1) due to the symmetric and asymmetric broadening of the dielectric loss peak which characterize the width of the distribution of the relaxation time. The Cole-Cole plot of the dielectric constant parallel to the molecule axis at 303 K is shown in Figure 3a. The fitting parameters are *a* = 0.82, *b* = 0.99, and *f*_R_ = 18.8 kHz. The plot gives a slightly asymmetric semicircle due to *a* being a little smaller than 1. The temperature dependence of the relaxation frequency, extracted by Equation (1), is presented in Figure 3b. Generally, the temperature dependence of the relaxation frequency can be described by the Arrhenius equation *f*_R_ ∝ exp(−*E_a_*/*k_b_T*), where *E_a_* is the activation energy, *k_b_* is the Boltzmann constant, and *T* is absolute temperature. The measured temperature dependence of the relaxation frequency followed the Arrhenius equation well with *E_a_* = 87.9 kJ·mol^−1^ as shown in Figure 3b.

**Figure 4.**
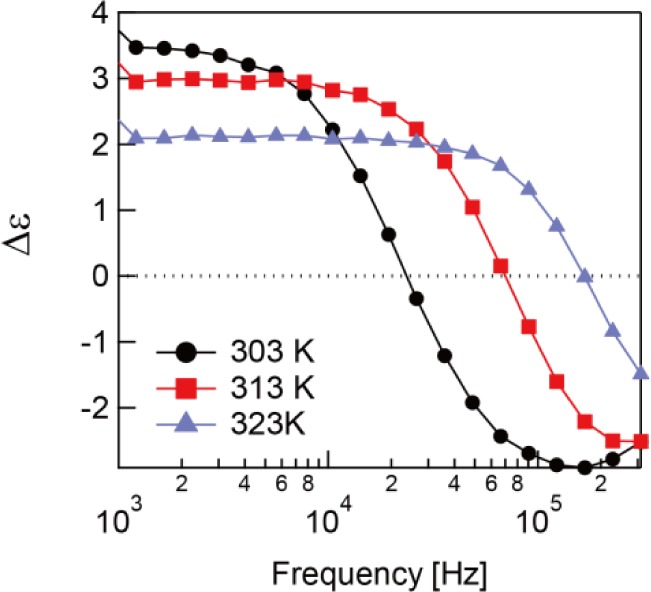
Frequency dependence of the dielectric anisotropy before polymerization.

A RM molecule has two components of its dipole vector longitudinal and transverse to its long axis. The orientational polarization occurs when the dipoles are rotationally fluctuated by the electric field, and the dielectric constants ε_‖_ = ε_‖_′ − ε_‖_″ and $\varepsilon_{⊥}={\varepsilon^{\prime }}_{⊥}-{\varepsilon^{\prime \prime }}_{⊥}$ are generated from the rotational fluctuations parallel and perpendicular to the molecule long axis, respectively. Since the relaxation process was only found with ε_‖_, the relaxation of the unpolymerized sample was attributed to the rotational fluctuation of the molecules around the molecule short axis called δ-relaxation [16]. The obtained activation energy is an acceptable value for the δ-relaxation of nematic liquid crystals [16,20]. The δ-relaxation generally occurs at lower frequency than β-relaxation which is caused by the rotational fluctuations of molecules around the molecule long axis [16,20]. This means the relaxation process with ε_⊥_ is supposed to show up in the higher frequency region. The asymmetric semicircle of the Cole-Cole plot means that the relaxation includes multi-relaxation processes. Therefore, the RM used in this study, which is mixture of several RMs, should have multi-relaxation processes. Since only ε_‖_ has the relaxation process in the measured frequency region, $\varepsilon^{\prime }{}_{⊥}$ become larger than ε_‖_′ in the high frequency region while ε_‖_′ is larger than $\varepsilon^{\prime }{}_{⊥}$ in the low frequency region. Hence, the sign of the dielectric anisotropy, Δε = ε_‖_ − ε_⊥_ changes from positive to negative at a specific frequency called the crossover frequency as can see in Figure 4. This is the typical behavior of DFLCs [18].

**Figure 5.**
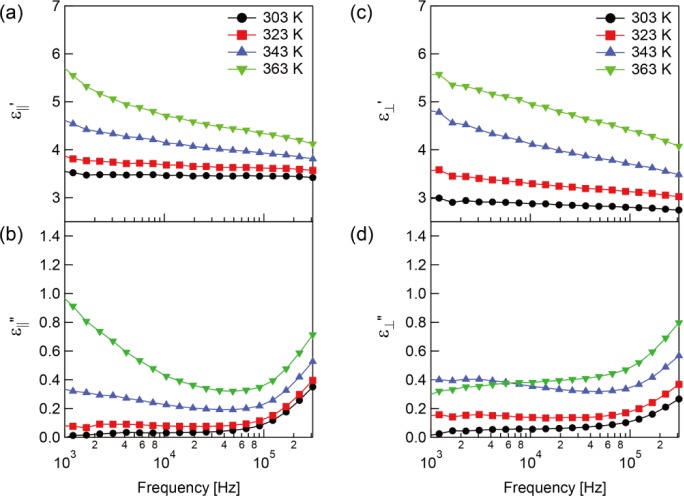
Measured complex dielectric constants after polymerization. The (**a**) real part and (**b**) imaginary part of complex dielectric constant parallel to the molecular long axis and the (**c**) real part and (**d**) imaginary part of complex dielectric constant perpendicular to the molecular long axis.

**Figure 6.**
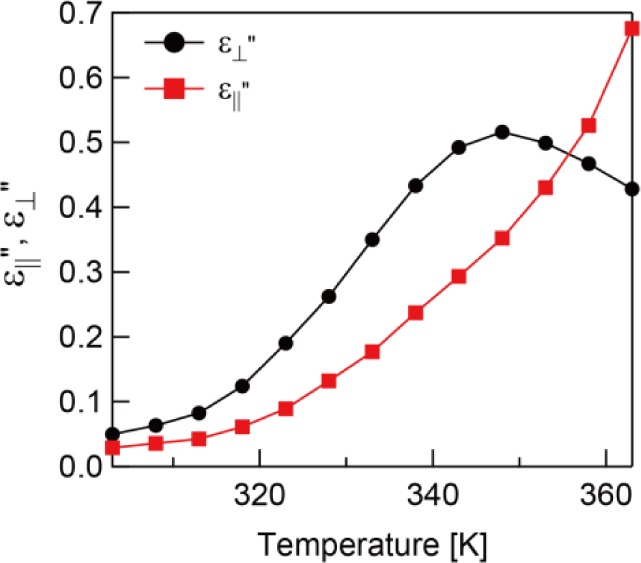
Temperature-dependent imaginary parts of the complex dielectric constants of the polymerized sample at 3 kHz.

The real parts of the complex dielectric constants of the sample after polymerization were lower than before polymerization for the entire measured frequency region (Figure 5a,c). While $\varepsilon^{\prime }{}_{⊥}$ of the unpolymerized sample does not vary with temperature, $\varepsilon^{\prime }{}_{⊥}$ of the polymerized sample does. This tendency can also be seen with ε_‖_′. In addition, the same temperature dependence was also found with ε_‖_″ and $\varepsilon^{\prime \prime }{}_{⊥}$ as shown in Figure 5b,d. Although there were no outstanding peaks with ε_‖_″ and $\varepsilon^{\prime \prime }{}_{⊥}$, weak peaks in $\varepsilon^{\prime \prime }{}_{⊥}$ were found at the low frequency region. The temperature dependence of the imaginary parts of the dielectric constants at 3 kHz is shown in Figure 6. Both ε_‖_″ and $\varepsilon^{\prime \prime }{}_{⊥}$ increase with increasing temperature as mentioned previously and $\varepsilon^{\prime \prime }{}_{⊥}$ has a peak indicating the existence of dielectric loss.

**Figure 7.**
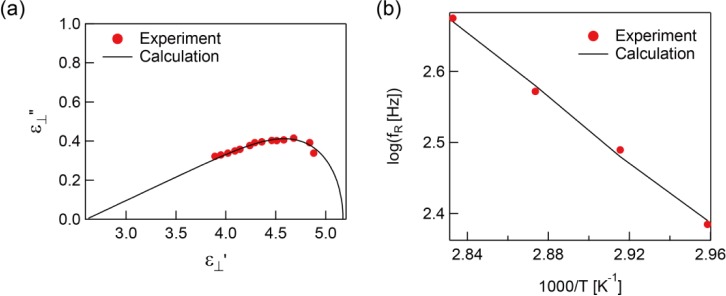
(**a**) The Cole-Cole plot of the polymerized sample at 343 K. (**b**) Temperature dependence of the relaxation frequencies of $\varepsilon^{\prime \prime }{}_{⊥}$ before polymerization. The filled circles are experimental data and the line is calculation results.

In Figure 7a, the Cole-Cole plot of $\varepsilon^{\prime \prime }{}_{⊥}$ is shown with the calculation from Equation (1) using fitting parameters *a* = 0.15, *b* = 0.95, and *f*_R_ = 308.7 Hz. The resultant plot is quite different from a symmetric semicircle due to the low value of *a* caused by a large relaxation time distribution. The temperature dependence of the relaxation frequency for $\varepsilon^{\prime \prime }{}_{⊥}$ is shown in Figure 7b. The result followed the Arrhenius equation similar to ε_‖_″ of the unpolymerized sample. The activation energy was calculated as 43.4 kJ·mol^−1^.

The lower dielectric constants of PRM can be attributed to the restriction of rotational fluctuations of PRM molecules because of the connection of each molecule leading to decreasing contribution of the orientational polarization. Generally, δ-relaxation is not able to activate with main chain liquid crystalline polymers in which mesogens are incorporated directly into a main chain [15,16]. Moreover, in case of side chain liquid, crystalline polymers in which mesogens are decoupled from a main chain by a spacer, δ-relaxation can only become active above the glass transition temperature, *T*_g_ [16,17,21]. Hence, δ-relaxation must not occur with our system where *T*_g_ = 372 K as measured by differential scanning calorimetry.

Increasing ε_‖_″ and $\varepsilon^{\prime \prime }{}_{⊥}$ with increasing temperature could be attributed to α-relaxation assigned to micro-Brownian motion of a main chain being active around *T*_g_. It is reported that α-relaxation can be observed with both ε_‖_″ and $\varepsilon^{\prime \prime }{}_{⊥}$ owing to random movement of micro-Brownian motion [21]. Since the temperature dependence of ε_‖_″ and $\varepsilon^{\prime \prime }{}_{⊥}$ observed in PRM is not observed in RM, the dependence should be an inherent characteristic of the polymeric system similar to the glass transition temperature. The dielectric constants at low frequency are slightly higher than at high frequency. It is believed that the α-relaxation peak will show up at the low frequency region at higher temperature.

The relaxation process indicated by a weak peak in $\varepsilon^{\prime \prime }{}_{⊥}$ is attributed to β-relaxation [15,17,21]. The β-relaxation is also observed in non-liquid crystalline polymeric systems arising from rotational fluctuations of localized parts of a main chain and/or side chains attached to a main chain. It should be mentioned that the relaxation time distribution of β-relaxation is quite large because of the occurrence of several relaxation processes. In the case of 4-[6-(Acryloyloxy)hexyloxy]benzoic acid=4-cyanophenyl ester, the fluctuations of the ester group near the main chain and fluctuations of the mesogen are reported [17,21]. In addition, the other materials mixed with the sample could broaden the dielectric loss peak as shown in Figure 7 (a). The obtained activation energy of the relaxation with $\varepsilon^{\prime \prime }{}_{⊥}$ is an acceptable value for β-relaxation [16,17,21].

**Figure 8.**
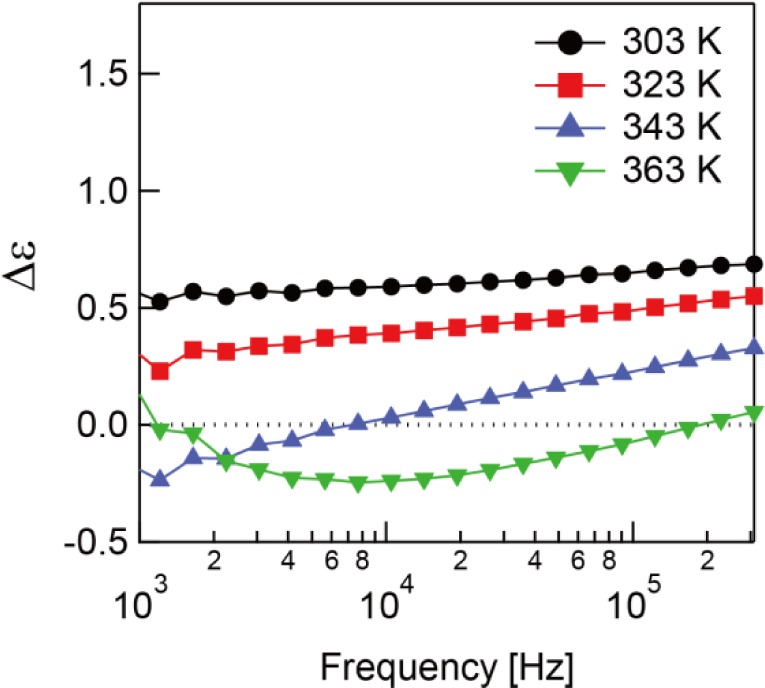
Frequency dependence of the dielectric anisotropy after polymerization.

In Figure 8, the dielectric anisotropy of the polymerized sample is shown. It is worth mention that the sign of the dielectric anisotropy changes from negative to positive above 338 K, which is the opposite behavior of the sample before polymerization. This dual-frequency behavior is given by the β-relaxation. Although the δ-relaxation dominates the system before polymerization, the β-relaxation takes precedence after polymerization. It is thought this phenomena only occurs with the RMs with large ε_⊥_ or with a large component of the dipole vector transverse to the long axis of the molecule.

## 4. Conclusions

The dielectric properties of the RM before and after polymerization were investigated. The RM showed the dielectric relaxation of the δ-relaxation arising from rotational fluctuation of the molecules around the molecule short axis. Consequently, the RM showed DFLC behavior. On the other hand, the PRM had the dielectric relaxation assigned to the β-relaxation generated from the rotational fluctuation of the molecules around the molecule long axis. The PRM also showed dual-frequency characteristics. However, the dielectric anisotropy, with increasing frequency, was observed to go from positive to negative before polymerization and negative to positive after polymerization.
